# Functionalized Non-vascular Nitinol Stent via Electropolymerized Polydopamine Thin Film Coating Loaded with Bortezomib Adjunct to Hyperthermia Therapy

**DOI:** 10.1038/s41598-017-08833-x

**Published:** 2017-08-25

**Authors:** Ludwig Erik Aguilar, Batgerel Tumurbaatar, Amin Ghavaminejad, Chan Hee Park, Cheol Sang Kim

**Affiliations:** 1 Department of Bionanosystem Engineering, Graduate School, Chonbuk National University, Jeonju City, Republic of Korea; 2 Department of Mechanical Design Engineering, Chonbuk National University, Jeonju City, Republic of Korea; 3 Power Engineering School, Mongolian University of Science and Technology, Ulaanbaatar, Mongolia; 4 Eco-friendly Machine Parts Design Research Center, Chonbuk National University, Jeonju City, Republic of Korea

**Keywords:** Drug delivery, Drug delivery

## Abstract

Gastrointestinal malignancies have been a tremendous problem in the medical field and cover a wide variety of parts of the system, (i.e. esophagus, duodenum, intestines, and rectum). Usually, these malignancies are treated with palliation with the use of non-vascular nitinol stents. However, stenting is not a perfect solution for these problems. While it can enhance the quality of life of the patient, in time the device will encounter problems such as re-occlusion due to the rapid growth of the tumor. In this study, we propose a functionalization technique using electropolymerization of polydopamine directly onto the nitinol stent struts for the combined application of hyperthermia and chemotherapy. The coating was characterized using FESEM, XPS, and FT-IR. Drug release studies show that facile release of the anticancer drug BTZ from the surface of the polydopamine-coated stent could be achieved by the dissociation between catechol groups of polydopamine and the boronic acid functionality of BTZ in a pH-dependent manner. The anti-cancer property was also evaluated, and cytotoxicity on ESO26 and SNU-5 cancer cell lines were observed. Our results suggest that the introduced approach can be considered as a potential method for therapeutic stent application.

## Introduction

In recent years, stents in non-vascular applications have been a standard treatment for relieving occluding tumors in the gastrointestinal (GI) system^1,2^. Malignant tumors in the GI tract can be found virtually anywhere in the system especially in the esophagus, duodenum, small intestine, colon, and rectum^3,4^. Such occlusion can create problems to the normal bodily functions of the GI, for example, the passage of food to the stomach, chime to the duodenum, and also important enzymes from the accessory organs of the GI such as the pancreas, gall bladder, and liver^5–7^. Occlusions in the upper GI can lead to the aggravation of the condition of the patient by reducing the nutrient absorption in the body. Malignant tumors are usually diagnosed late and one of the primary palliative treatment is stenting. Non-vascular stents that are usually made of nitinol alloy can push back the tumor occlusion, create patency in the orifice, and maintain normal passage of food or other biological substances.

Non-vascular stents can lead to the reopening of the occlusion in the GI but the said treatment doesn’t come without fallbacks. One problem that we encounter is the re-occlusion of the tumor into the stent strut due to the rapid growth rate of the tumor. Since the caloric and nutritional intake of the patient are somehow returned to normal, it creates a negative feedback loop that contributes to the main problem. One solution is to maintain or reduce the tumor size by functionalizing the stent struts with a polymeric layer and loading it with an anti-cancer drug^1^.

Electropolymerization is a coating technique that utilizes an electrochemical deposition of conductive monomers to polymerize directly onto the substrate or electrode and subsequently creating a thin film which we can control the thickness by controlling the number of cycles we ran^8^. One example of a conductive molecule is dopamine^9^. Dopamine is a natural biomolecule that can be found inside the body as a neurotransmitter^10^. Dopamine can be polymerized on the surface of conductive substrates either by classical self-polymerization (s-pdopa) or by electropolymerization (e-pdopa)^11,12^. In contrast to self-polymerization, electropolymerization of dopamine can provide more control over the properties of the coated film. By polymerizing dopamine onto the stent, we can utilize its catechol moiety to bind to the boronic acid functional group of the anti-cancer drug bortezomib (BTZ)^9,13^. BTZ was chosen as the drug of choice mainly due to the ability of the drug to bind to the catechol moiety of polydopamine and the link is pH cleavable and could be controlled by adjusting the pH of its surrounding^14^. However, there are still adverse pharmacokinetic effects of BTZ, these includes nonspecific binding to proteins, dose-limiting toxicities, and rapid hepatic clearance from blood that can be avoided if the drug can be linked to a polymeric carrier^15,16^. The GI has a dynamic pH environment, especially in the duodenum. When the patient is in the fed state the pH becomes more acidic with a mean pH value of < 5.5 and during the fasting state, the mean pH value becomes > 5.5^17^. Using this information, we can use the pH gradient as a triggering mechanism for drug release.

Another strategy to maintain the tumor volume and avoid re-occlusion is with the use of hyperthermia therapy^18^. Nitinol is inherently ferromagnetic due to the presence of Nickel in the alloy (49%). Once we apply an external alternating magnetic field, the stent can increase its temperature applying heat to the tumor. Hyperthermia therapy has been studied already *in-vitro* and *in-vivo* by many researchers and promising results has been noted^19–23^. Increasing the tumor temperature to the therapeutic level of 40–45 °C can lead to cytotoxicity of the cancer cells due to the inefficient heat regulation of the tumor. Fast angiogenesis leads to a faulty vasculature system that hinders proper heat regulation inside the cancerous tissue^24^. The excess heat can destroy the cancer cells by damaging the DNA in the nucleus and promoting cell death. The effects of hyperthermia depend on the exact heating capability of the material being used. Proper heat dosage and exposure time are needed to have therapeutic effects in the body.

The combination of pH-dependent release of bortezomib and hyperthermia can maintain the tumor volume thus avoiding re-occlusion. In this study, we investigate the combination of these two treatment modalities and their potential to improve stenting. We partnered a controlled drug release system through the pH sensitive binding between bortezomib (BTZ) and catechol groups of polydopamine and an effective hyperthermia therapy by subjecting the nitinol stent under the external alternating magnetic field to increase its temperature to hyperthermic conditions. The coating was characterized using FESEM, XPS, and FTIR. Drug release studies show that facile release of the anticancer drug BTZ from the surface of the polydopamine-coated stent could be achieved due to the dissociation between catechol groups of polydopamine and the boronic acid functionality of BTZ. The anti-cancer property of the stent was also evaluated and cytotoxicity on ESO26 and SNU-5 cancer cells were observed.

## Results

### Electropolymerization of Polydopamine onto the Nitinol Stent

**Figure 1:**
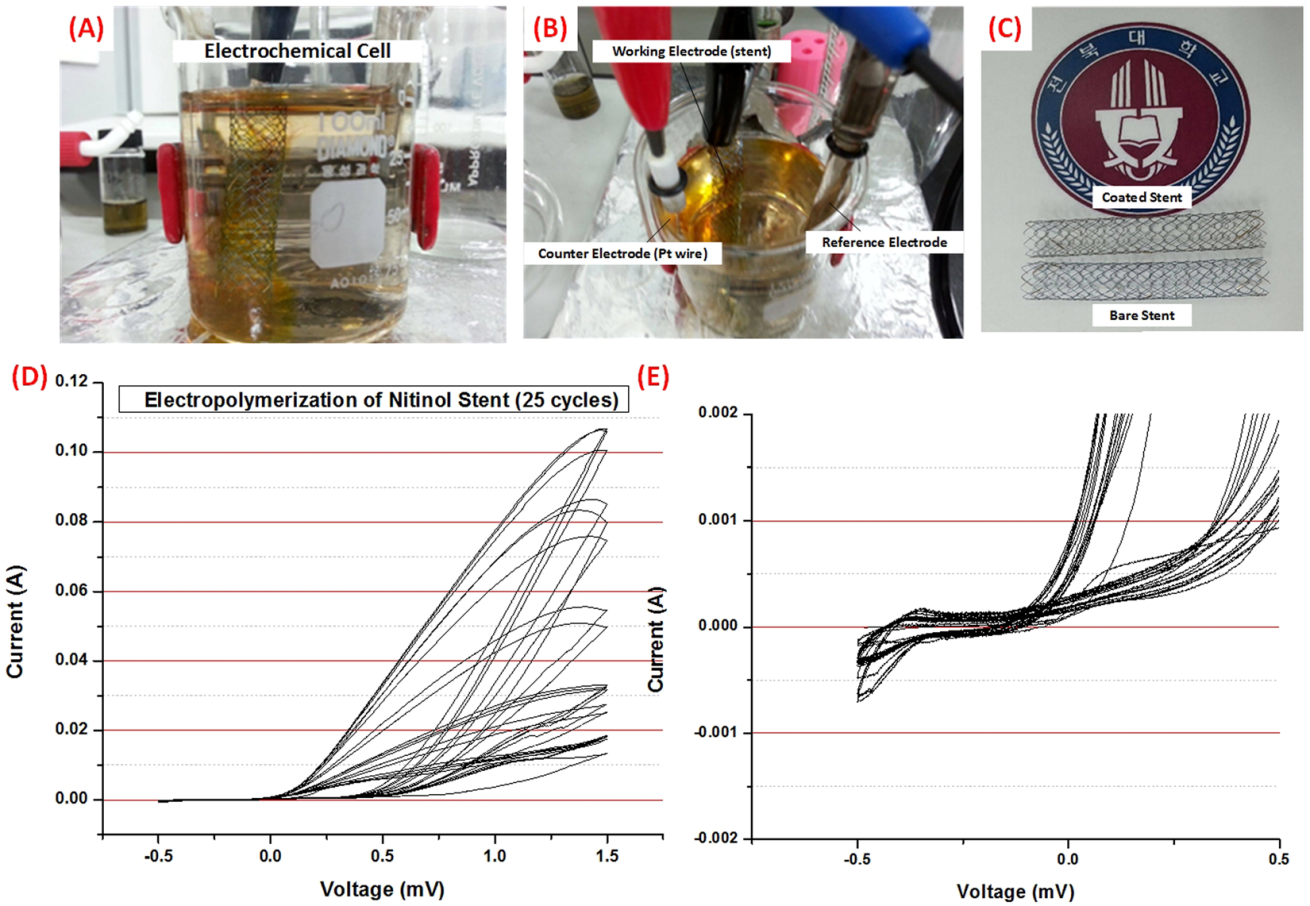
Electropolymerization procedure (**A**,**B**) Electrochemical cell showing the stent as the working electrode. (**C**) Finished 60 mm nitinol stent (top) after 25 cycles of electropolymerization compared to bare nitinol stent (bottom). (**D**) Cyclic voltammetry graph of the electropolymerization procedure at 25 cycles (**E**) Cyclic voltammetry graph showing the reduction of polydopamine.

The process of electropolymerization of polydopamine can be seen on Fig. 1A,B where it can be noted that the redox reaction of dopamine leads to polymerization directly onto the stent. The color of the solution changes as the cycles of electropolymerization started, from clear to light brown in color. The deposition of the coating can be determined by the decrease in conductivity of the working electrode as seen in Fig. 1D, where the conductivity of the working electrode is inversely proportional to the thickness of the polydopamine coating^9^. The reduction of dopamine in the solution can be verified also by the CV graph in Fig. 1E, where anodic peaks can be noted, indicative of redox couples of dopamine/dopamine quinone, also the peak area decreased every after scanning as a result of the deposition of the polydopamine thin film on the nitinol stent strut. The flow of the current is hindered by the increasing growth of the polydopamine coating which is expected due to the electrical insulating effects of the polymer. Figure 1C shows the end product of the electropolymerization process where the polydopamine coating can be seen by the change in color of the stent sample, from being a bluish tint to light brown.

### Validation of the Polydopamine Coating under FESEM, AFM, ATR-FTIR, and XPS

**Figure 2:**
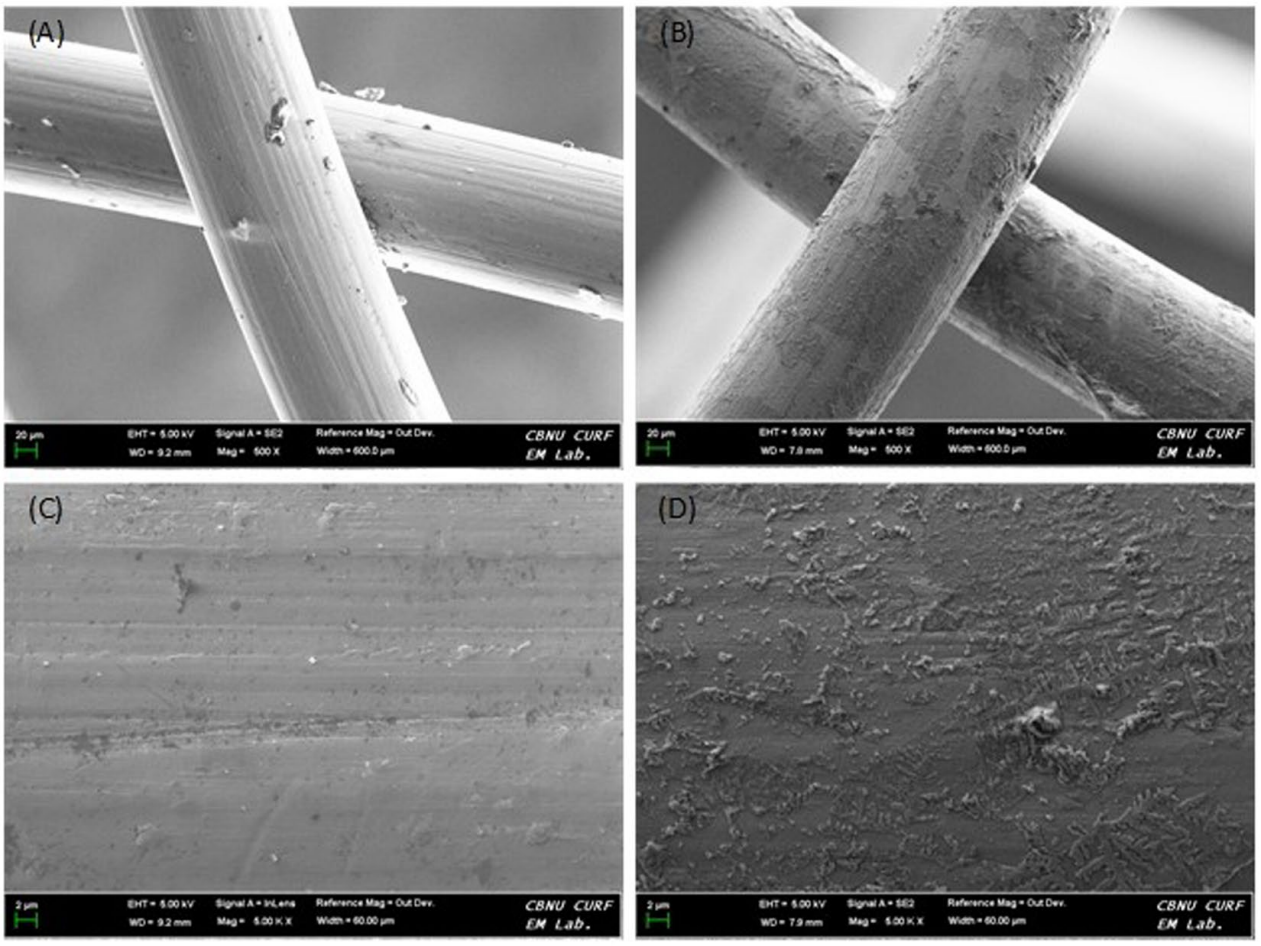
FESEM images of nitinol stent struts (**A**) bare nitinol at low magnification (**B**) e-pdopa/nitinol at low magnification (**C**) bare nitinol surface high magnification (**D**) e-pdopa/nitinol surface high magnification.

FESEM imaging was conducted to visibly validate the formation of the e-pdopa coating on the nitinol stent strut. On Fig. 2A,B we can see the difference between the coated and bare nitinol stent struts, the e-pdopa stent struts has a rougher surface due to the presence of a coating while the bare nitinol stent struts have a smoother surface appearance. Upon closer inspection under higher magnification on Fig. 2C,D, we can notice the difference in surface morphology and roughness of the two stent samples, a thin film has been formed on the surface of the e-pdopa-nitinol specimen. Referring to the FESEM images, we can visually verify the coating of polydopamine onto the nitinol stent struts. The e-pdopa coating can now be used as a base for functionalizing the stent surface by exploiting the catechol groups of polydopamine. Loading the polydopamine coating with a borate containing anticancer drug (bortezomib) is now possible with their 1,2-benzenediol (catechol) moieties for enabling a pH-dependent drug delivery towards the cancer cells.

**Figure 3:**
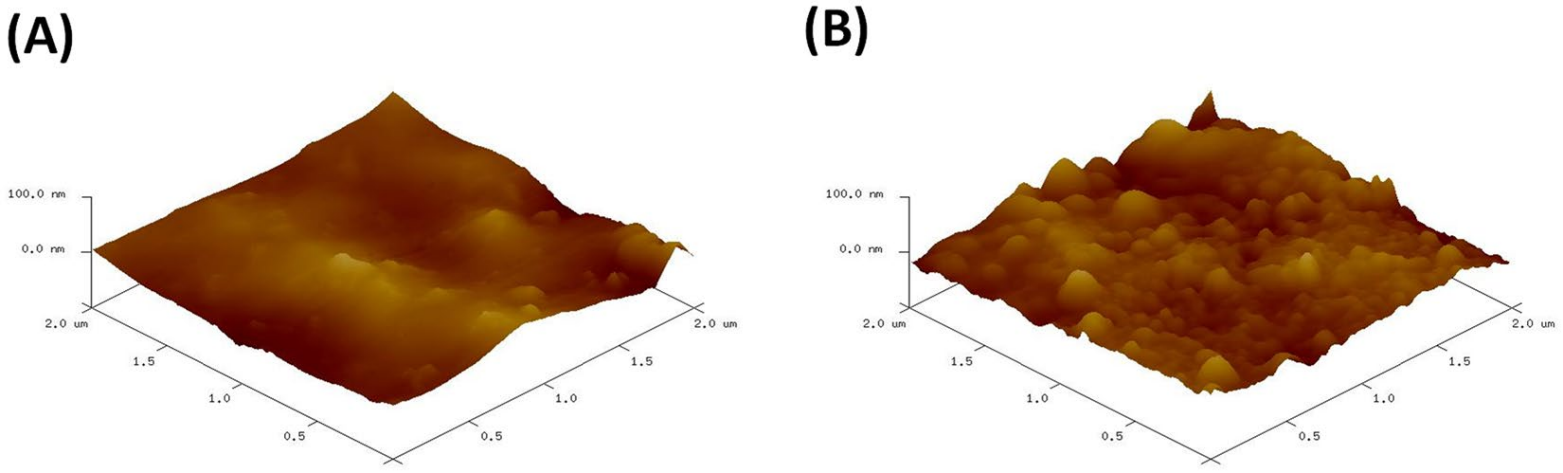
Atomic Force Microscopy (AFM) of the surface of (**A**) pure nitinol (**B**) e-pdopa-nitinol.

The surface roughness indicated that the modification of the nitinol substrate is successful, to further validate the difference in surface morphology and corroborate the FESEM results, Atomic Force Microscopy (AFM) was conducted. In Fig. 3A, the surface roughness of the pure nitinol sample can be found to be uniform and smooth with few indentation and ridges to be seen. In Fig. 3B, the surface of the substrate has changed dramatically after the coating process, with nanoscale peaks and ridges formed on the surface of the nitinol sample.

**Figure 4:**
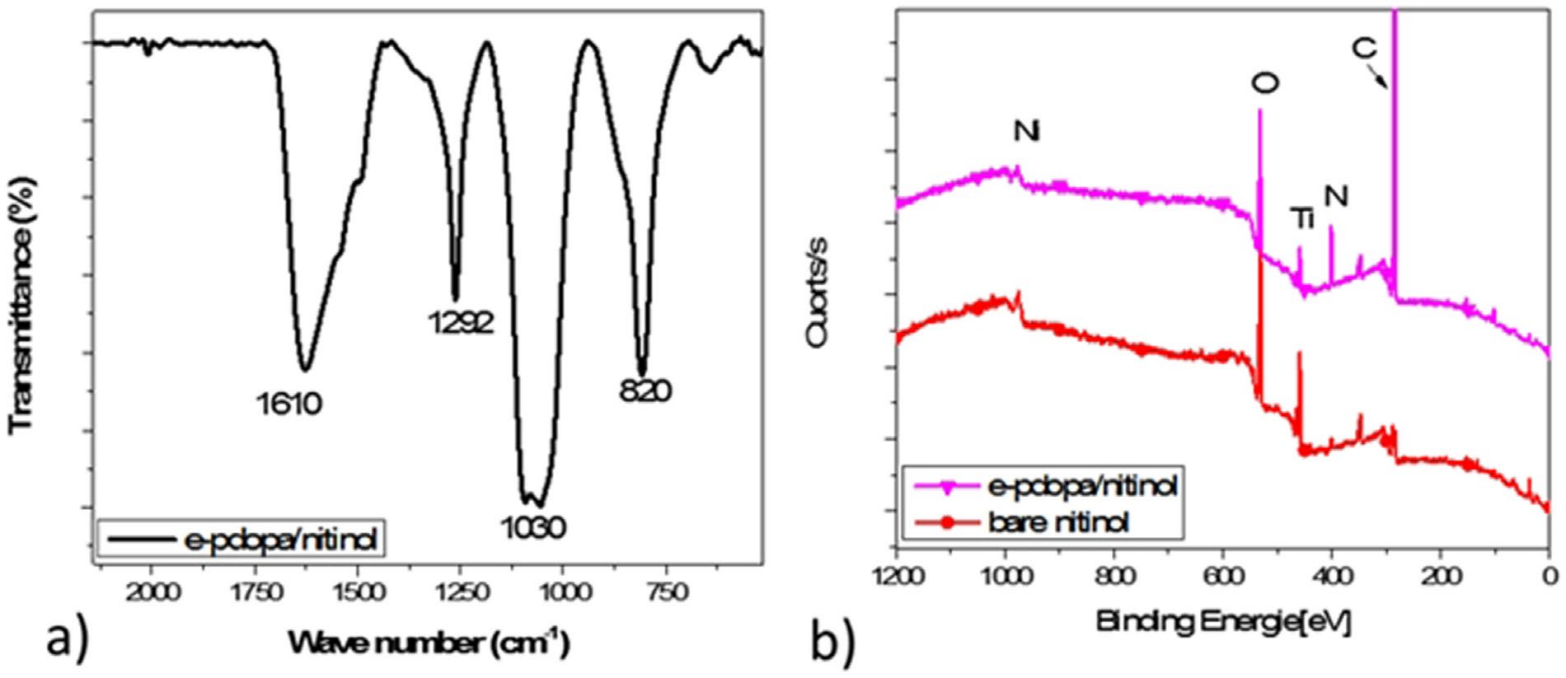
(**a**) Attenuated Total Reflection Fourier Transform Infrared Spectra (ATR-FT-IR) and (**b**) X-ray Photon Spectroscopy (XPS) of bare nitinol, e-pdopa/nitinol, and e-pdopa-btz/nitinol.

The formation of polydopamine on the surface of nitinol stent was confirmed by performing the Attenuated Total Reflection-Fourier Transform Infrared spectra (ATR-FTIR) (see Fig. 4a). The spectrum shows two peaks at 1610 cm^−1^ and 1292 cm^−1^ corresponding to the stretching vibration of the C=C and the phenolic C–OH of polydopamine, respectively. Two vibrational modes at 1030 and 820 cm^−1^ can also be assigned to the amine group of dopamine^25,26^. Furthermore, the surface chemical composition of bare nitinol and e-pdopa-nitinol were characterized by XPS spectroscopy (Fig. 4b). The XPS results of e-pdopa/nitinol in comparison with the bare stent showed that the polydopamine coating generated the characteristic carbon (phenolic) and nitrogen peaks (amine group). Indicating that the surface of the nitinol specimen has been covered with polydopamine film.

### Drug Loading Efficiency of Bortezomib

Loading the BTZ drug onto the e-pdopa/stent followed a facile approach by dissolving BTZ into distilled H_2_O/DMSO with a 10:1 ratio and subsequently submerging the stent samples into the solution. The pH of the solution was increased to 9.0 to ensure the binding between catechol moiety and the boronic acid drug. It should be mentioned that the possibility of complexation between BTZ and catechol groups has been deeply studied in our previous works^14,27,28^.

We determined the loading capacity of the coated stents by measuring the remaining BTZ on the supernatant after the drug loading process of 24 hours. The initial drug concentration in the solution was determined by measuring 500 µg/ml of BTZ solution and analyzing it under high-performance liquid chromatography, and after the drug loading process the supernatant was analyzed again and shown a decreased BTZ concentration of 250 ± 20 µg/ml. Approximately 250 ± 20 µg/ml of BTZ were loaded into the stent surface. With this data and using equation no.1 for calculating entrapment efficiency, we determined that the e-pdopa coating has a 50 ± 4% entrapment efficiency.

### Heating Capability Studies of the Non-vascular Nitinol Stent under Alternating Magnetic Field

**Figure 5:**
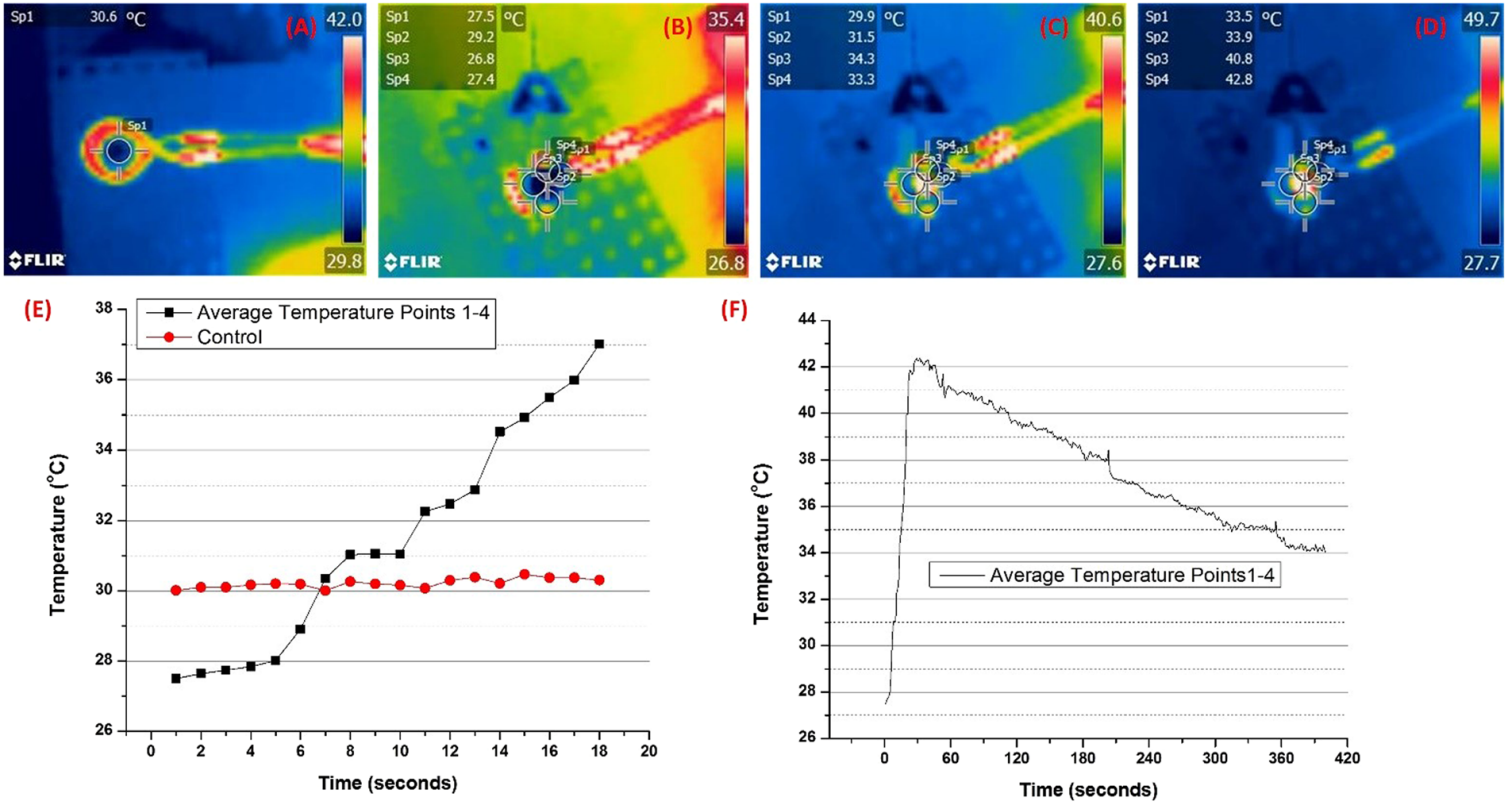
Heat dissipation study of the e-pdopa-btz/nitinol stent under Alternating Magnetic Field (AMF) taken using FLIR camera (**A**) control (pure acrylic acid hydrogel) (**B**–**D**) heating spectra of the stent at different time points (**E**–**F**) Stent thermal change overtime.

In adjunct to the chemotherapy drug that was loaded onto the e-pdopa coating, we also evaluated the hyperthermia capability of the nitinol stent. As seen in Fig. 5B–D, the heating capability of the stent under the alternating magnetic field can be visualized using infrared imaging. Figure 5B shows the initial time when the AMF was turned on with the solenoid coil increasing in temperature while the middle of the coil where the stent was placed remains relatively colder compared to the coil with a mean temperature of 27 °C. Figure 5C demonstrate the point when the stent started to heat up with a visible infrared glow in the middle of the coil, Fig. 5D is when the AMF was turned off showing the coil losing its infrared glow and starting to reduce to room temperature but the stent still remains heated with some parts of the gel reaching 42 °C. The required hyperthermia temperature of 40–45 °C for the optimal therapeutic effects can be reached in a short amount of time of 12 secs and can be maintained for almost 5 mins as seen on Fig. 5E,F. Heating the cancer tumor as fast as possible is beneficial in achieving effective treatment due to the inability of the cancer cells to self-regulate its local temperature, and also due to the inefficient cellular stacking and vasculature formation of the cancerous tumor. The mainstream heating platform for cancer treatment nowadays is with the use of magnetic nanoparticles with the aid of alternating magnetic field. But their capability to maintain heat and reach the required therapeutic temperatures are limited by the concentration of magnetic component available^29^. However, in-stent hyperthermia, the high amount of Nickel in the nitinol alloy allows the efficient heating of the tumor at a relatively short amount of time, as noted in the heating studies performed in this research. Nitinol has a fast and abrupt temperature change capability under alternating magnetic field induction. Reports of stent hyperthermia’s safety and feasibility in the treatment of esophageal cancer has been investigated in several works as well^22,29^. However, the main problem that arises is the necrosis in the area where heating has been excessive. With the use of the anticancer drug BTZ, the combined effects of Hyperthermia and Chemotherapy can be utilized by reducing the heating dosage to less than 45 °C, potentially limiting the adverse effects of overheating in the local tissue.

### pH-Dependent *In-vitro* Drug Release Studies of Bortezomib

**Figure 6:**
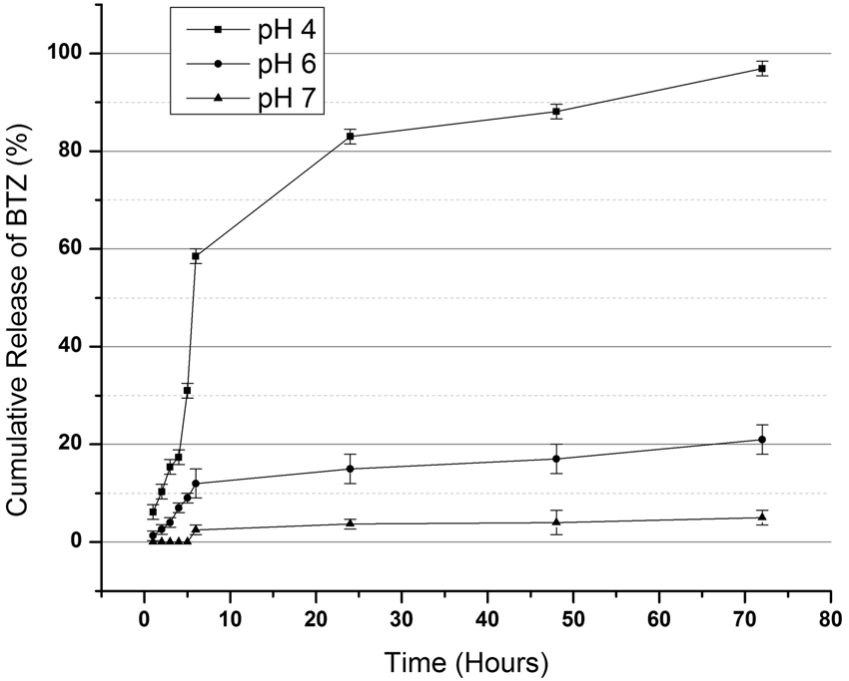
Bortezomib (BTZ) release studied under three pH conditions (pH 4.0, 6.0, and 7.0) that can be found in the gastrointestinal system.

The premise of pH change in the duodenum can be exploited for the treatment by using the acidic pH as the drug release trigger for BTZ into the target tumor^30^. Also, one example of a pH gradient inside the body is in the duodenum. This part of the GI has a dynamic pH that changes whenever the person is on fed or fasting state. The pH of the duodenum becomes acidic when fresh bolus from the stomach is passed through. We investigated the drug release profile of the e-pdopa-btz/nitinol under three pH conditions to mimic the natural pH of different sites in the GI system, and as seen in Fig. 6, the drug release behavior of BTZ is pH dependent. The catechol-boronic acid bonding between the polydopamine coating and BTZ can be cleaved by adjusting the pH to an acidic condition, in this case at pH 4.0, the release rate of BTZ is relatively slow at the first 4 hours noted only at 17%. After constant exposure to acidic conditions, BTZ can be noted to be released in a linear trend at 30% at 5 hours, 58% at 6 hours, 83% at 24 hours, 88% at 48 hours and 96% at 72 hours. This is advantageous as we wouldn’t like to release all the drug payload at a relatively short time. The pH environment of the duodenum goes back to above 6.0 when the bolus has completely passed through the duodenum at approximately 4 hours. This pH change can only be noted at this part of the upper GI system and when the same functionalization process is done to other non-vascular stents such as esophageal or colonic stents, this kind of release profile can’t be achieved and the BTZ drug payload can remain in the coating for a longer time period, as evidenced by the lesser drug release profile in pH 6 and 7. The BTZ payload can remain intact in the coating making it usable for a longer period of time if we employ this functionalization technique to non-pH changing parts of the GI system.

### *In-vitro* Biocompatibility Studies

**Figure 7:**
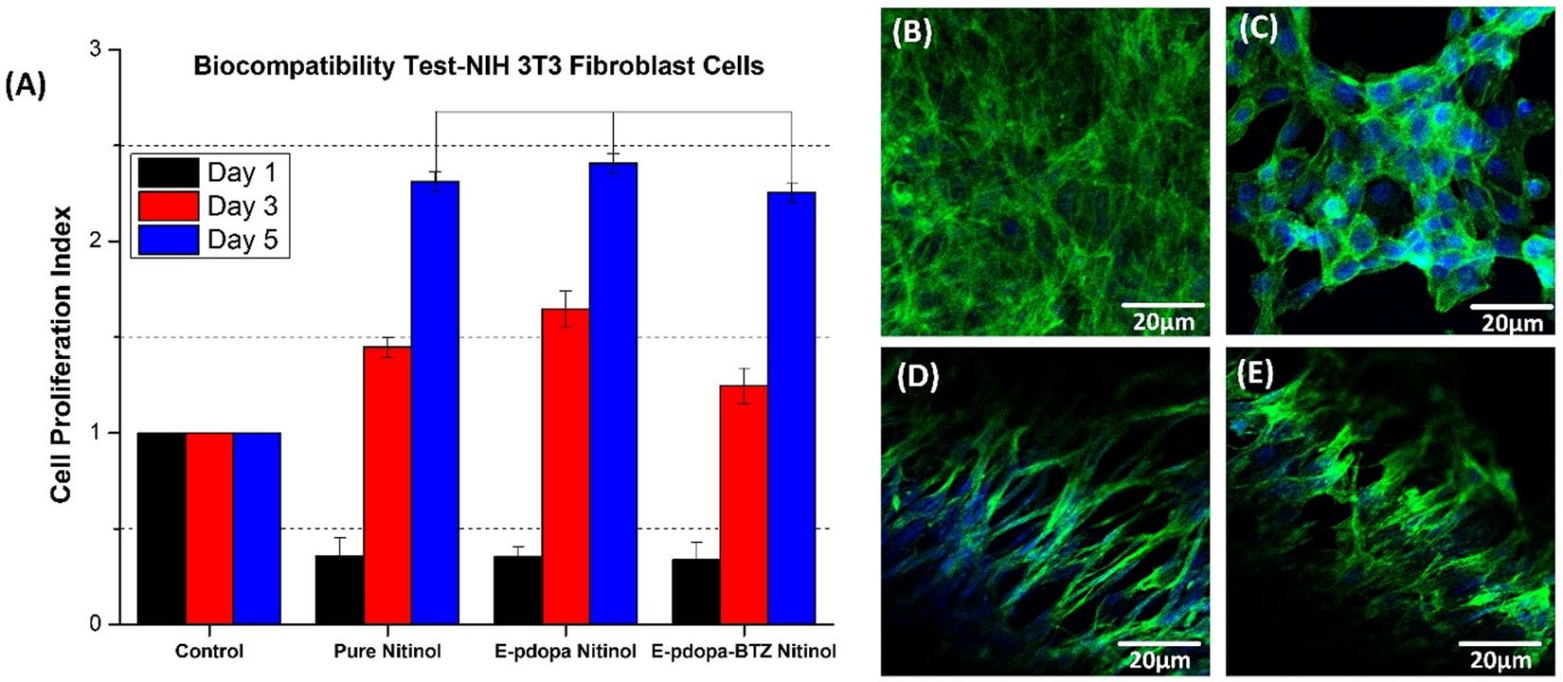
Biocompatibility studies using NIH 3T3 fibroblast cells using CCK8 assay (**A**) Cell proliferation Index of the cells; Confocal Imaging (DAPI and Actin Green) (**B**) Control (**C**) Bare Nitinol (**D**) e-pdopa/nitinol (**E**) e-pdopa-btz/nitinol. (Data are expressed as mean ± SD, *n* = 3. *p < 0.05) (one way ANOVA post hoc Tukey test).

The next thing we have to evaluate is the biocompatibility of the coating, the proliferation rate of the NIH-3T3 fibroblast cells on pure nitinol, e-pdopa/nitinol, and e-pdopa-btz/nitinol can be seen in Fig. 7A. No significant differences noted on the proliferation rate of the fibroblast cells on all of the samples after 5 days of culture, cell proliferation indexes were noted at 2.3, 2.4, and 2.2 for pure nitinol, e-pdopa/nitinol, and e-pdopa-btz/nitinol, respectively. However, the cell proliferation index on Day 1 is reduced at 0.3 on all of the samples due to the inability of the initially loaded cells to adhere properly, but cell proliferation was noted on the CCK8 assay once the cells adhered to the surface. The morphology of the cells was further evaluated using DAPI and actin-green staining to visualize how the cells would grow on different substrates. The growth pattern of the cells shown morphological differences among all of the substrates. On Fig. 7C, cell agglomeration can be seen as the growth pattern of the fibroblast cells. This could be due to the inefficient adherence of the cells onto the metal substrate. On the other hand, the e-pdopa/nitinol substrate have better cell adhesion and proliferation compared to pure nitinol as evidenced by the longer actin growth on each cell, Fig. 7D,E. The same goes with the BTZ loaded substrate but with slightly lower proliferation rate as shown in Fig. 7A. The polydopamine coating could help in passivizing the surface of the nitinol making it slightly more biocompatible than the pure nitinol substrate.

### *In-vitro* Anticancer Studies

**Figure 8:**
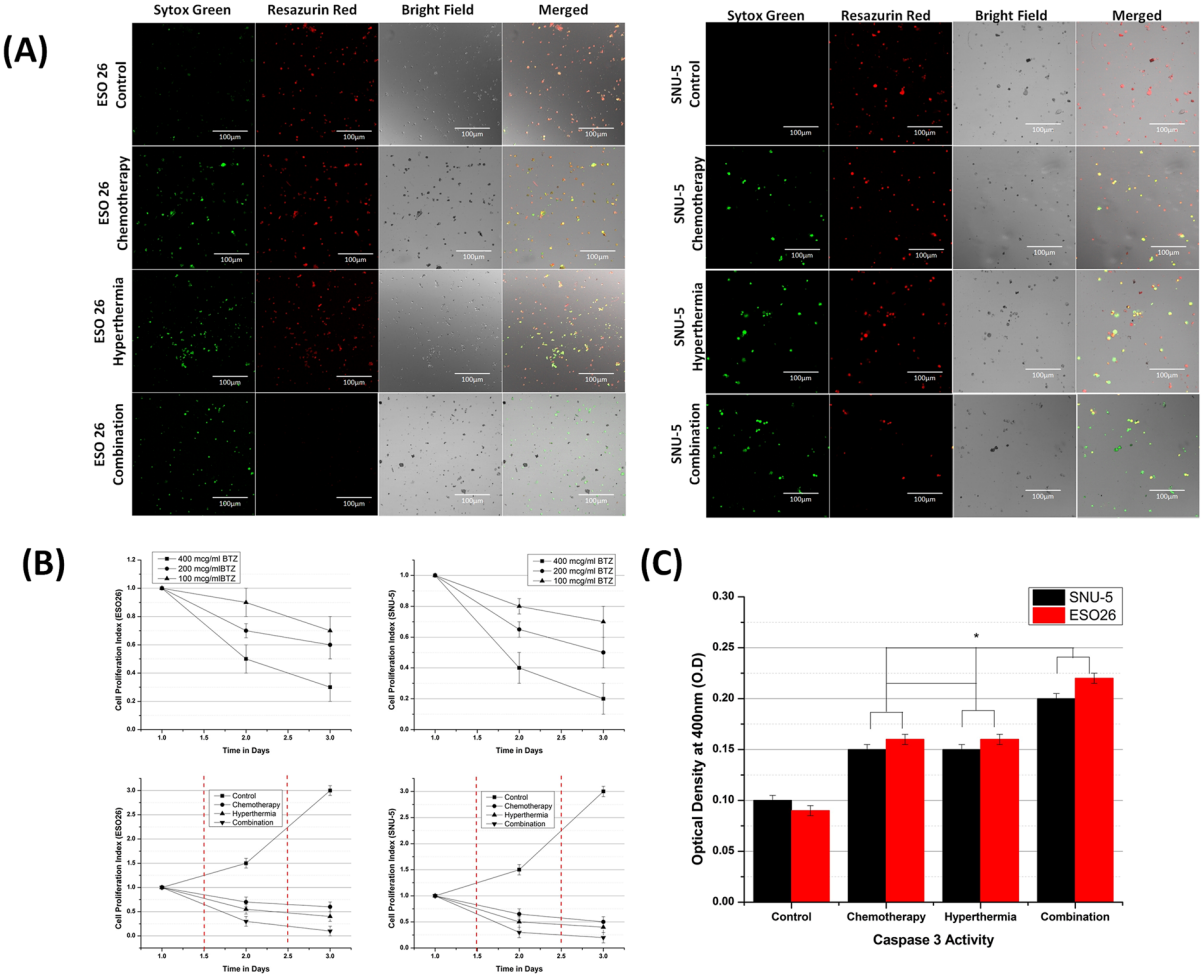
*In vitro* anti-cancer studies using ESO26 and SNU-5 human cancer cell lines (**A**) Live/Dead Assay (Red = Live, Green = Dead/Injured) (**B**) Cell proliferation Index (Broken red line indicates the time when AMF was switched ON) (**C**) *in vitro* CASPASE3 activity assay. (Data are expressed as mean ± SD, *n* = 3. *p < 0.05) (one way ANOVA post hoc Tukey test).

Once we have validated the biocompatibility and drug loading capability of the coating, we proceeded to evaluate the anticancer property of the e-pdopa-btz/nitinol and the potential application on reducing the re-occlusion scenario. The cell lines that has been selected were ESO26 (esophageal) and SNU-5 (gastric) human cancer cell lines. On Fig. 8A, the live/dead assay data shows that the red fluorescence (live cells) on combination treatment vs. hyperthermia and free BTZ are far less in frequency. Also, the hyperthermia treatment shows more live cells compared to the combination treatment but exhibited more cell death vs. the free BTZ group. The combination group shows almost all of the cells are in dead/injured state as evidenced by the dominant green fluorescence on both cell lines. The combination of hyperthermia and BTZ could lead to a higher cytotoxicity effects with almost zero percent of the cells alive after 3 days of culturing for both ESO26 and SNU-5 cell lines. The cell proliferation index, Fig. 8B, also concurs with the live/dead assay results showing us the day by day reduction of the CPI, compared to the control, all of the groups that received treatment show a reducing trend on CPI but with the combination treatment proving to be the most effective in reducing the ESO26 and SNU-5 cancer cell count with 0.1 ± (0.1) and 0.2 ± (0.1) CPI scores after each hyperthermia treatment after day 1 and day 2 respectively. Different dosages of free BTZ were also studied (400 µg/ml, 200 µg/ml, and 100 µg/ml) with an exposure time of 4 hours, we can denote that increasing cell death is comparative to the increased dosage of BTZ. The increased temperature at 43 °C from 37 °C can create increased oxygenation inside the cells and could also lead to higher sensitivity to anticancer drugs^31^. To determine the apoptotic level of for each treatment group we have conducted an *in-vitro* CASPASE3 assay. The results in Fig. 8C shows that the combination treatment has the highest CASPASE3 activity at 0.2 ± (0.05) and 0.22 (0.05) OD for SNU-5 and ESO26 cell lines respectively, followed by Hyperthermia group and Chemotherapy group, having the same results of 0.15 ± (0.05) OD (SNU-5) 0.16 ± (0.05) (ESO26) OD. CASPASE3 is an indicator protein that is involved in activating CASPASE 8 and 9 also known as the caspase cascade or caspase activated apoptosis pathway.

## Discussion

**Figure 9:**
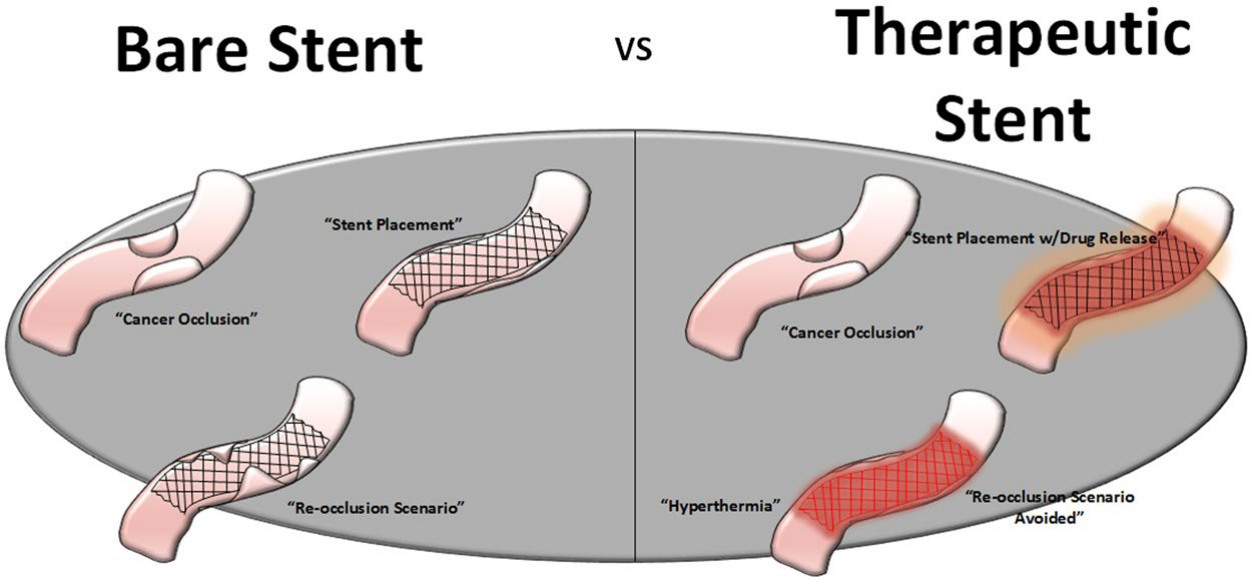
Schematics of Therapeutic stent advantage vs bare stent, where re-occlusion scenario can be avoided by using the combined effects of hyperthermia therapy and chemotherapeutic drug release.

Gastrointestinal malignancies are mostly treated with palliation to ensure proper nutrition for patients. However, non-vascular stenting has several fallbacks as it is prone to re-occlusion after implantation. In Fig. 9, we explain the schematics of the re-occlusion scenario, where the bare stent is rendered ineffective by the ingrowth of the malignant tumor. In the case of therapeutic stenting, we are functionalizing the stent with a polymeric layer that can release anti-cancer drugs to maintain the tumor volume, also by partnering it with hyperthermia therapy via induction through alternating magnetic field we can increase the effects of the anti-cancer drug.

Electrochemistry using cyclic voltammetry has been known to be used in a wide variety of applications and characterization of materials^32–34^. Such as determining corrosion rate of metals and quantifying compounds in a given solution^34–36^. For our application, we have used the intrinsic property of the naturally occurring phenolic molecule dopamine with its ortho isomeric benzenediols and amine group to form a catechol moiety containing functional thin film layer in the nitinol substrate^37,38^. These phenols and phenolic like compounds can form films in the working electrode and could hinder the quantifying sensitivity of the electrode as the peak distances tend to decrease^37^. However, this phenomenon is advantageous in a certain aspect of surface functionalization, as we would like to create a thin film where we can load anticancer compounds that can be bonded using the catechol groups of the polydopamine.

Characterizations of the coating given in this study show the successful formation of the polydopamine onto the nitinol substrate. This kind of technique has been reported also in our previous work^13,14^. Where we explain the advantage of electropolymerization versus self-polymerization of dopamine under alkali conditions, papers also have been published using polydopamine as a drug and protein carrier for practical uses in the biomedical field. It has been used in vascular stenting for increasing vascular re-endothelialization by binding vascular endothelial growth factors in the polydopamine film formed by electropolymerization. In this work, we now further demonstrate the flexibility of application of polydopamine by using it as a platform for anticancer drug delivery on non-vascular stents. The drug loading efficacy also shows that a high amount of drug can be loaded in the coating, the surface functionalization of the nitinol stent was able to utilize the reactions with catechol moiety that served as the binding site for the BTZ drug. Bortezomib is the first therapeutic proteasome inhibitor that can halt the spread of cancer cells found in solid malignant tumors. Multiple mechanisms in the intracellular environment must be involved, but the most probable pathway for the anticancer property of bortezomib is the proteasome inhibition, it may prevent degradation of pro-apoptotic factors, permitting activation of programmed cell death in neoplastic cells dependent upon suppression of pro-apoptotic pathways. The ability of our stent to release the drug payload depending on the pH of the environment is one of the benefits of using e-pdopa coating, as the catechol-boronic acid bond is cleavable by reducing the pH to less than 5.0. The drug release study has shown us that the drug is stable under pH conditions of 6.0 and 7.0 where it is mostly retained in the e-pdopa coating. The GI is a dynamic environment when it comes to pH, it constantly fluctuates depending on the condition of the person. When a person has an occlusion in the duodenum, where the pH changes depending on whether the patient is on a fed or fasting state, the pH-dependent release of BTZ can be used in such environment. Furthermore, chemotherapy could be enhanced by using an adjuvant therapy such as hyperthermia. In our investigation, we demonstrated the ability of the nitinol stent to increase in temperature by subjecting it to an alternating magnetic field. The increased temperature of 40.0–45.0 °C can hinder DNA recombination in the nucleus and promote protein denaturation that could lead to apoptosis^39,40^.

We have also shown that the coating in a pH neutral condition is stable and NIH-3T3 cells can proliferate in the e-pdopa-btz-nitinol substrate as shown in Fig. 7D,E. Nickel is known to be mildly toxic to biological tissues, and several passivation techniques must be done to ensure the safety and feasibility of the metal in biomedical applications. Nitinol having 49% nickel must undergo passivation such as creating an oxide layer to provide a protective interface between the biological tissue and alloy. Polymeric coatings had been employed also to passivize several types of metals for biomedical devices. PTFE coating is widely used in metallic implants due to its low dielectric constant. A biologically derived polymer such as polydopamine can also be a protective layer to passivize and also functionalize any metallic substrate to ensure safety in implantation^13^.

Most reports that show magnetic hyperthermia treatment showing lesser anticancer properties for the hyperthermia group compared to free anticancer drugs^31^. This could be due to the fact that the material that they have used are made up of magnetic nanoparticles with slower heating capabilities and fast heat loss mechanisms^29^. The heating of nitinol is abrupt and could shock the cells making it more cytotoxic than free drug treatment. It is known that an abrupt and prolonged exposure to hyperthermia temperatures can lead to higher cytotoxic effects to the cancer cells and potentially have better therapeutic effects^41^.

## Experimental

### Materials

Dopamine Hydrochloride is obtained from Sigma Aldrich, Bortezomib from (LC Laboratories, USA), Tris, NaCl, KCl, were all obtained from Samchun Chemicals (South, Korea), 60 mm nitinol stents were given by Taewoong Medical (South, Korea), 0.02 inch thickness Nitinol foil for characterization and testing were purchased from Alfa Aesar (South, Korea). All aqueous solutions were prepared with ultrapure water purified with a Milli-Q UV-Plus water purification system (Millipore, Bedford, MA). The water had a resistivity of >1018 MΩ cm^−1^.

### Electropolymerization Process

Multiple scanning cyclic voltammetry was used for electrochemical polymerization of dopamine. Figure 1D,E shows the typical CV of dopamine polymerized at a concentration of 1 mg/mL Tris buffer saline solution (TBS, pH 7.4) on a nitinol stent at 1.5 to −1.5 A. The solution was prepared first via degassing distilled water with N_2_ gas for 30 mins. After which dopamine HCl was weighed and added to the solution. Appropriate amounts of Tris (3 mg/ml), NaCl (8 mg/ml), and KCl (0.22 mg/ml) were also added and dissolved using a magnetic stirrer under nitrogen bubbling. The solution was then placed in a 100 ml beaker that served as the electrochemical cell. The electrodes that were used were Pt wire (counter), Ag/AgCl (reference), Nitinol Stent (working). The parameters for the reduction of dopamine onto the stent was set at Voltage −1.5 mV (initial), 0.5 mV (middle), −1.5 mV (final) and Scanning rate was set at 50 µV/s. After each cycle, the area under the curve in the voltammogram decreases as a result of the deposition of polydopamine onto the working electrode^9,13^. After 25 cycles (50 segments), the amount of polymer that had been deposited led to an almost complete insulation of the working electrode.

### Loading of Bortezomib in the polydopamine-coated stent

First, the drug was weighed at 5.0 mg and dissolved in a 10 ml water/DMSO mixture (V/V = 10:1) with a resulting drug concentration of 500 µg/ml. The pH of the drug solution was then adjusted to 9.0 with drops of NaOH 0.1 M. The e-pdopa stent was then fully submerged for 24 hours at room temperature. The drug loading efficiency was measured via High-performance liquid chromatography (HPLC) (retention time of 6.633; wavelength 263 nm) (JASCO, USA) by measuring the amount of BTZ left in the supernatant after the loading process and comparing it to a number of drugs present before the drug loading procedure.

The entrapment efficiency was then calculated using the following equation:

1 $${\boldsymbol{E}}{\boldsymbol{n}}{\boldsymbol{t}}{\boldsymbol{r}}{\boldsymbol{a}}{\boldsymbol{p}}{\boldsymbol{m}}{\boldsymbol{e}}{\boldsymbol{n}}{\boldsymbol{t}}\,{\boldsymbol{E}}{\boldsymbol{f}}{\boldsymbol{f}}{\boldsymbol{i}}{\boldsymbol{c}}{\boldsymbol{i}}{\boldsymbol{e}}{\boldsymbol{n}}{\boldsymbol{c}}{\boldsymbol{y}}\,({\boldsymbol{ \% }})=\frac{{\boldsymbol{a}}{\boldsymbol{m}}{\boldsymbol{o}}{\boldsymbol{u}}{\boldsymbol{n}}{\boldsymbol{t}}\,{\boldsymbol{o}}{\boldsymbol{f}}\,{\boldsymbol{d}}{\boldsymbol{r}}{\boldsymbol{u}}{\boldsymbol{g}}\,{\boldsymbol{e}}{\boldsymbol{n}}{\boldsymbol{t}}{\boldsymbol{r}}{\boldsymbol{a}}{\boldsymbol{p}}{\boldsymbol{e}}{\boldsymbol{d}}}{{\boldsymbol{a}}{\boldsymbol{m}}{\boldsymbol{o}}{\boldsymbol{u}}{\boldsymbol{n}}{\boldsymbol{t}}\,{\boldsymbol{o}}{\boldsymbol{f}}\,{\boldsymbol{d}}{\boldsymbol{r}}{\boldsymbol{u}}{\boldsymbol{g}}\,{\boldsymbol{u}}{\boldsymbol{s}}{\boldsymbol{e}}{\boldsymbol{d}}}\times {\bf{100}}$$

### Characterization of the Functionalized Stent Coating

The morphology of the coating was observed using Field Emission Scanning Electron Microscope (Carl Zeiss Supra 40VP). The samples were sputter coated under argon in order to make them electrically conductive. The excitation voltage used to capture the images was set at 2 kV.

Fourier transform Infrared spectroscopy data was obtained using the Spectrum-GX FTIR spectrometer (PerkinElmer Co., USA). The scanning range was set at 500–4000 cm^−1^ with a resolution of 1 cm^−1^. The elemental composition and surface state of the samples was checked using X-ray photoelectron spectroscopy (XPS, AXIS-NOVA, Kratos, Inc.) with an Al Kα irradiation source.

### Alternating Magnetic Field Induced Stent Heating Studies

The stent heat dissipation study was done by embedding a 60 mm stent sample in a poly(acrylamide) gel with 70% water content to mimic the physiological modulus of the body. The poly(acrylamide) gel was synthesized in a 20 ml glass mold that contain stent by mixing deionized water (18.37 g), monomer (1.60 g acrylamide, 22.5 mmol vinyl groups), crosslinker (43.1 mg N,N′-methylene bis-(acrylamide), 0.56 mmol vinyl groups) and initiator (66.5 mg 2,2′-Azobis(2-methylpropionitrile) 0.36 mmol) respectively.

The heating properties and heat dissipation of the stent onto the surrounding acrylamide gel were observed and measured using a real-time observation using FLIR C2 camera and software. The alternating magnetic field system (OSH-120-B, OSUNG HITECH, Republic of Korea) generator used in the study has a power output of 12.5 kA m^−1^ and frequency of 270 kHz, the system has a copper solenoid coil with a diameter of 30 mm and has 6 loops and is cooled using a water circulator.

### *In-vitro* Drug Release Test

The *in-vitro* drug release test was carried out to test the pH-dependent release of bortezomib in the e-pdopa-stent. Three PBS solutions with different pH that mimics the environmental pH of the GI system was used. The pH of the PBS was adjusted by directly tittering HCl to the PBS solutions. PBS with pH of 4.0,6.0, and 7.0 was used for the drug release test. The e-pdopa stents were then submerged in the PBS solutions and 1 ml aliquots were then taken at specific time intervals. The PBS was then replaced with pre-warmed fresh PBS solutions to maintain the media concentration. The aliquots were then analyzed using High-performance liquid chromatography (HPLC) (retention time of 6.633; wavelength 263 nm) (JASCO, USA). The experiment was done in triplicates and the reported values were the averaged values obtained.

### *In vitro* Biocompatibility test

For the biocompatibility test, the material used was an e-pdopa-nitinol foil to effectively seed the adherent cells. Fibroblast (NIH-3T3) cells were cultured at 37 °C under 5% CO_2_ in Dulbecco’s Modified Eagle Medium (DMEM, GIBCO) supplemented with 10% fetal bovine serum (FBS, GIBCO) and 1% penicillin-streptomycin. The cells were grown on the foils for 1, 3, and 5 days and the proliferation was investigated using Dojindo’s cell counting kit-8. The e-pdopa-nitinol foil was sterilized using Ultraviolet irradiation overnight and placed on 48 well plates. 100 µl of NIH-3T3 (fibroblast) cell suspension (5,000 cells/well for samples without drug content and 5,000 cells/well for samples with drug content). DMEM/high glucose (supplemented with 10% FBS and 1% penicillin-streptomycin) was dispensed in a pre-incubated 48 well plates containing foil samples and allowed to incubate in a humidified atmosphere of 5% CO_2_ at 37 °C for the designated time. The morphological study of the cells was observed using DAPI and actin-green staining and was subsequently viewed under confocal microscopy LSM-510 (Carl-Zeiss, Germany).

### *In vitro* evaluation of Anti-cancer properties of the e-pdopa-btz/stent

A comprehensive test was done to evaluate the effectiveness of the e-pdopa-stent hyperthermia in cancer cytotoxicity using ESO26 (esophageal adenocarcinoma) (Sigma-Aldrich) and SNU-5 (gastric carcinoma) cancer cell lines (Korean Cell Line Bank, KCLB, Korea). A cck8 assay using Dojindo Cell Counting Kit was done in determining the day by day cell proliferation index (CPI). The cell proliferation index was calculated using the following equation:

2 $${\boldsymbol{Cell}}\,{\boldsymbol{Proliferation}}\,{\boldsymbol{Index}}={{\boldsymbol{N}}}_{{\boldsymbol{D}}}/{{\boldsymbol{N}}}_{{\boldsymbol{D}}1=1}$$

whereas: N_D_ = Cell number on day D,

N_D1=1_ = Cell number on day 1

Live/Dead assay was done to determine the distribution of the live/dead/injured cells using Resazurin-red and Sytoxgreen dyes (Thermo Fischer, USA). The cells were gathered and analyzed using confocal microscopy LSM 510 Meta (Carl Zeiss, Germany). Apoptosis study was done using ApoTarget® caspase3/CPP32 colorimetric protease assay (Invitrogen, CA, USA). The cells were lysed and centrifuged at 10,000 rpm for 3 mins using a MicroCL 21 R microcentrifuge (Thermo-Fisher, USA) and the surfactant was analyzed using a microplate reader at 400 nm wavelength. The conditions that were studied were Hyperthermia, Free Drug at different set doses of 400 µg/ml, 200 µg/ml, and 100 µg/ml, and Combination Therapy. The hyperthermia conditions were set at 43 °C. The alternating magnetic field used was set at 270 kHz and the exposure time maintained at 12 secs maintaining the temperature therapeutic range for 5 mins.

### Statistical analysis

Data were compared using one-way ANOVA, using an OriginLab® software. Data are expressed as means ± SD of measurements (*p < 0.05 and **p < 0.01)

## References

[1] Kim SY (2014). Paclitaxel-eluting nanofiber-covered self-expanding nonvascular stent for palliative chemotherapy of gastrointestinal cancer and its related stenosis. Biomed Microdevices.

[2] Nicholson T (2000). Other uses of non-vascular stents. Hosp Med.

[3] Laurini JA, Carter JE (2010). Gastrointestinal Stromal Tumors A Review of the Literature. Arch Pathol Lab Med.

[4] Wong HH, Chu P (2012). Immunohistochemical features of the gastrointestinal tract tumors. Journal of gastrointestinal oncology.

[5] Lee JE (2015). Impact of Carcinomatosis on Clinical Outcomes after Self-Expandable Metallic Stent Placement for Malignant Gastric Outlet Obstruction. PloS one.

[6] Li J (2016). Covered versus Uncovered Self-Expandable Metal Stents for Managing Malignant Distal Biliary Obstruction: A Meta-Analysis. PloS one.

[7] Zorron PL (2015). Endoscopic stenting for inoperable malignant biliary obstruction: A systematic review and meta-analysis. World journal of gastroenterology.

[8] Luo XL, Cui XYT (2011). Electrochemical deposition of conducting polymer coatings on magnesium surfaces in ionic liquid. Acta Biomater.

[9] Wang JL (2014). Electropolymerization of dopamine for surface modification of complex-shaped cardiovascular stents. Biomaterials.

[10] Beaulieu JM (2005). An Akt/beta-arrestin 2/PP2A signaling complex mediates dopaminergic neurotransmission and behavior. Cell.

[11] Park J (2014). Polydopamine-Based Simple and Versatile Surface Modification of Polymeric Nano Drug Carriers. Acs Nano.

[12] Zhu D (2016). Docetaxel (DTX)-loaded polydopamine-modified TPGS-PLA nanoparticles as a targeted drug delivery system for the treatment of liver cancer. Acta Biomater.

[13] GhavamiNejad A (2015). Immobilization of silver nanoparticles on electropolymerized polydopamine films for metal implant applications. Colloid Interfac Sci.

[14] GhavamiNejad A (2015). Mussel-Inspired Electrospun Smart Magnetic Nanofibers for Hyperthermic Chemotherapy. Adv Funct Mater.

[15] Wu S (2013). pH-Responsive Drug Delivery by Amphiphilic Copolymer through Boronate–Catechol Complexation. ChemPlusChem.

[16] Su J, Chen F, Cryns VL, Messersmith PB (2011). Catechol Polymers for pH-Responsive, Targeted Drug Delivery to Cancer Cells. J Am Chem Soc.

[17] Dressman JB (1990). Upper Gastrointestinal (Gi) Ph in Young, Healthy-Men and Women. Pharmaceut Res.

[18] GhavamiNejad, A., SamariKhalaj, M., Aguilar, L. E., Park, C. H. & Kim, C. S. pH/NIR Light-Controlled Multidrug Release via a Mussel-Inspired Nanocomposite Hydrogel for Chemo-Photothermal Cancer Therapy. *Sci Rep-Uk* **6**, 10.1038/srep33594 (2016). 10.1038/srep33594 PMC5028867 27646591

[19] Dahring H, Grandke J, Teichgraber U, Hilger I (2015). Improved Hyperthermia Treatment of Tumors Under Consideration of Magnetic Nanoparticle Distribution Using Micro-CT Imaging. Mol Imaging Biol.

[20] Kim YJ, Ebara M, Aoyagi T (2013). A Smart Hyperthermia Nanofiber with Switchable Drug Release for Inducing Cancer Apoptosis. Adv Funct Mater.

[21] Luo Y, Dahmardeh M, Chen X, Takahata K (2015). A resonant-heating stent for wireless endohyperthermia treatment of restenosis. Sensor Actuat a-Phys.

[22] Zhou JM (2009). Hyperthermia by a nitinol stent in an alternating magnetic field: Safety and feasibility in rabbit esophageal cancer. Prog Nat Sci.

[23] Aguilar LE, GhavamiNejad A, Park CH, Kim CS (2017). On-demand drug release and hyperthermia therapy applications of thermoresponsive poly-(NIPAAm-co-HMAAm)/polyurethane core-shell nanofiber mat on non-vascular nitinol stents. Nanomedicine: Nanotechnology, Biology and Medicine.

[24] De Palma GD (2016). *In Vivo* Assessment of Tumor Angiogenesis in Colorectal Cancer: Role of Confocal Laser Endomicroscopy. Digest Liver Dis.

[25] Thakur VK (2012). Novel polymer nanocomposites from bioinspired green aqueous functionalization of BNNTs. Polym Chem-Uk.

[26] GhavamiNejad A, Kalantarifard A, Yang GS, Kim CS (2016). *In-situ* immobilization of silver nanoparticles on ZSM-5 type zeolite by catechol redox chemistry, a green catalyst for A(3)-coupling reaction. Micropor Mesopor Mat.

[27] Sasikala ARK (2015). A smart magnetic nanoplatform for synergistic anticancer therapy: manoeuvring mussel-inspired functional magnetic nanoparticles for pH responsive anticancer drug delivery and hyperthermia. Nanoscale.

[28] Sasikala Arathyram Ramachandra Kurup, Unnithan Afeesh Rajan, Yun Yeo-Heung, Park Chan Hee, Kim Cheol Sang (2016). An implantable smart magnetic nanofiber device for endoscopic hyperthermia treatment and tumor-triggered controlled drug release. Acta Biomaterialia.

[29] Liu J (2013). Local hyperthermia for esophageal cancer in a rabbit tumor model: Magnetic stent hyperthermia versus magnetic fluid hyperthermia. Oncol Lett.

[30] Aguilar LE (2015). Electrospun polyurethane/Eudragit (R) L100-55 composite mats for the pH dependent release of paclitaxel on duodenal stent cover application. Int J Pharmaceut.

[31] Kumar CSSR, Mohammad F (2011). Magnetic nanomaterials for hyperthermia-based therapy and controlled drug delivery. Adv Drug Deliver Rev.

[32] Chrzescijanska E, Wudarska E, Kusmierek E, Rynkowski J (2014). Study of acetylsalicylic acid electroreduction behavior at platinum electrode. J Electroanal Chem.

[33] Fernandez C (2015). Pharmaceutical Electrochemistry: the Electrochemical Oxidation of Paracetamol and Its Voltammetric Sensing in Biological Samples Based on Screen Printed Graphene Electrodes. Int J Electrochem Sc.

[34] Manjunatha JG, Swamy BEK, Gilbert O, Mamatha GP, Sherigara BS (2010). Sensitive Voltammetric Determination of Dopamine at Salicylic Acid and TX-100, SDS, CTAB Modified Carbon Paste Electrode. Int J Electrochem Sc.

[35] Mielech-Lukasiewicz K, Puzanowska-Tarasiewicz H, Niedzielko A (2011). Electrooxidation of Some Antifungal Agents and Their Square-Wave Voltammetric Determination in Cosmetics and Pharmaceutics. Anal Lett.

[36] Susmel S, Comuzzi C (2015). 5-Phenyl-dipyrromethane and 5-(4-pyridyl)-dipyrromethane as modular building blocks for bio-inspired conductive molecularly imprinted polymer (cMIP). An electrochemical and piezoelectric investigation. Rsc Adv.

[37] Torriero AAJ, Luco JM, Sereno L, Raba J (2004). Voltammetric determination of salicylic acid in pharmaceuticals formulations of acetylsalicylic acid. Talanta.

[38] Wang ZH, Liu XL, Wu BW, Wang FP, Lu XQ (2012). Voltammetric Determination of Salicylic Acid by Molecularly Imprinted Film Modified Electrodes. Int J Polym Anal Ch.

[39] Issels RD (2008). Hyperthermia adds to chemotherapy. Eur J Cancer.

[40] Calderwood SK, Ciocca DR (2008). Heat shock proteins: Stress proteins with Janus-like properties in cancer. Int J Hyperther.

[41] Mallory M, Gogineni E, Jones GC, Greer L, Simone CB (2016). Therapeutic hyperthermia: The old, the new, and the upcoming. Crit Rev Oncol Hemat.

