# Publisher Correction: Functionalized Non-vascular Nitinol Stent via Electropolymerized Polydopamine Thin Film Coating Loaded with Bortezomib Adjunct to Hyperthermia Therapy

**DOI:** 10.1038/s41598-018-19724-0

**Published:** 2018-01-18

**Authors:** Ludwig Erik Aguilar, Batgerel Tumurbaatar, Amin Ghavaminejad, Chan Hee Park, Cheol Sang Kim

**Affiliations:** 1 Department of Bionanosystem Engineering, Graduate School, Chonbuk National University, Jeonju City, Republic of Korea; 2 Department of Mechanical Design Engineering, Chonbuk National University, Jeonju City, Republic of Korea; 3 Power Engineering School, Mongolian University of Science and Technology, Ulaanbaatar, Mongolia; 4 Eco-friendly Machine Parts Design Research Center, Chonbuk National University, Jeonju City, Republic of Korea

Correction to: *Scientific Reports* 10.1038/s41598-017-08833-x, published online 25 August 2017

The version of this Article previously published omitted Chan Hee Park as a corresponding author. The correct corresponding authors for this Article are Cheol Sang Kim & Chan Hee Park. Correspondence and request for materials should be addressed to chskim@jbnu.ac.kr or biochan@jbnu.ac.kr. This error has now been corrected in the HTML and PDF versions of the Article.

