# Publisher Correction: Functionalized Non-vascular Nitinol Stent via Electropolymerized Polydopamine Thin Film Coating Loaded with Bortezomib Adjunct to Hyperthermia Therapy

**DOI:** 10.1038/s41598-018-22365-y

**Published:** 2018-03-06

**Authors:** Ludwig Erik Aguilar, Batgerel Tumurbaatar, Amin Ghavaminejad, Chan Hee Park, Cheol Sang Kim

**Affiliations:** 1 Department of Bionanosystem Engineering, Graduate School, Chonbuk National University, Jeonju City, Republic of Korea; 2 Department of Mechanical Design Engineering, Chonbuk National University, Jeonju City, Republic of Korea; 3 Power Engineering School, Mongolian University of Science and Technology, Ulaanbaatar, Mongolia; 4 Eco-friendly Machine Parts Design Research Center, Chonbuk National University, Jeonju City, Republic of Korea

Correction to: *Scientific Reports* 10.1038/s41598-017-08833-x, published online 25 August 2017

An error occurred after publication resulting in the inadvertent omission of the Article and Volume numbers from the PDF version of this Article. This error has now been corrected in the PDF version of the Article; the HTML version was correct from the time of publication.

